# The Treatment of Laryngeal Tuberculosis

**Published:** 1905-02-11

**Authors:** 


### THE TREATMENT OF LARYNGEAL TUBERCULOSIS.

The frequency with which the larynx becomes affected in consumptive patients, and the distress which the ensuing condition may cause, render the subject worthy of careful study; and especially so as an idea seems to prevail that local treatment is of little use. Of course, general hygienic treatment is essential, but there are patients who go down hill even in sanatoria, while a still greater number are unable to undergo sanatorium treatment at all. For such patients a great deal may be done by means of local therapeutics. Mr. Harold Barwell, who is very positive on this point, in a contribution to the current number of the *Edinburgh Medical Journal* classifies the methods into three groups, namely, those which are merely palliative, others which combat the associated catarrh and secondary infection, and, thirdly, those which directly attack the tuberculous disease. Palliative treatment may be necessary where there is marked dysphagia. Cocaine may be used, preferably in a laryngeal spray producer and shortly before meals; the weakest effective solution must be employed, and it should only be applied by the medical attendant or a well-trained nurse. Eucaine may be used instead, but it appears to be less certain, while it has the same disadvantages as cocaine. Morphine hydrochloride may be insufflated half-an-hour before meals in doses of $\frac{1}{16}$ grain diluted with 2 grains of starch. Irrespective of their mental effect these drugs have one most deleterious action—they produce a marked loss of appetite, the results of which are disastrous to consumptive patients. A much better local anæsthetic than the foregoing is orthoform. Three to five grains are given as an insufflation. There is no toxic action, and the administration may be entrusted to the patient. The anæsthetic effect lasts from 12 to 24 hours, but the drug will not act through unbroken epithelium. Cough may be satisfactorily relieved by means of a linctus of heroin $\frac{1}{12}$ grain to the drachm of equal parts of glycerine and water. For laryngeal catarrh steam inhalations are of great value, especially the common one of Friar's balsam; an alkaline spray is beneficial when there is much secretion of muco-pus, or crusts, or débris overlying ulcers in the larynx. Remedial measures consist of topical applications of powerful drugs and direct surgical interference. Drugs are applied with a cotton-wool mop—a brush is very inferior both as to cleanliness and efficiency. The mop is introduced under inspection with the mirror, the patient himself holding out his tongue; mere painting on of the drug is not sufficient, it must be thoroughly rubbed into the affected part. The frequency of application should depend on the resulting reaction; the intervals may vary from one to seven days. Cocaine is seldom required after the first time, and should be dispensed with whenever possible, because of the bad effects mentioned above. Of all pigments lactic acid is the most effective. Strong solutions should be used *e.g.* 40 or 50 per cent. The application is painful, and the soreness sometimes persists for 12 hours or so; an insufflation of orthoform, given after the painting, will usually relieve the pain. Mr. Barwell generally uses a mixture containing 50 per cent. of lactic acid, 7 per cent. of formalin, and 10 per cent. of carbolic acid in water, reducing the strength when the ulcer begins to heal, or if there is too much reaction. The carbolic acid is a local anæsthetic, and pain is decidedly less than when lactic acid is used alone. Under such treatment ulcers frequently heal and moderate infiltration is cured, but no great effect is produced upon massive infiltration lying beneath unbroken epithelium; for such cases, and also for those where there is deep or extensive ulceration, surgical measures are required. These consist in the main of curetting ulcers and granulations on the posterior wall, ventricular bands, and epiglottis, and removing portions of large arytenoid or epiglottic swellings with cutting forceps, under cocaine anæsthesia. Hæmorrhage, as a rule, is very slight, and the larynx heals with great rapidity. A nebuliser with menthol in parolein will combat any inflammatory reaction. So potent are these surgical measures that Mr. Barwell has even seen improvement obtained by such means in the out-patient department of a London hospital.

